# Cost Saving Potential of an Early Detection of Atrial Fibrillation in Patients after ICD Implantation

**DOI:** 10.1155/2018/3417643

**Published:** 2018-08-14

**Authors:** Thomas Reinhold, Roberto Belke, Tino Hauser, Christian Grebmer, Carsten Lennerz, Verena Semmler, Christof Kolb

**Affiliations:** ^1^Institute for Social Medicine, Epidemiology and Health Economics, Charite-Universitätsmedizin Berlin, Luisenstrasse 57, 10117 Berlin, Germany; ^2^Biotronik, Woermannkehre 1, 12359 Berlin, Germany; ^3^German Heart Centre Munich, Department of Electrophysiology, Faculty of Medicine, Technische Universität München, Munich, Germany

## Abstract

Atrial fibrillation (AF) is a relevant comorbidity in recipients of implantable cardioverter-defibrillators (ICD). Latest generation single-chamber ICD allow the additional sensing of atrial tachyarrhythmias and, therefore, contribute to the early detection and treatment of AF, potentially preventing AF-related stroke. The present study aimed to measure the impact on patient-related costs of this new ICD compared to conventional ICD. A Markov model was developed to simulate the long-term incidence of stroke in patients treated with a single-chamber ICD with or without atrial sensing capabilities. The median annual cost per patient and its difference, the number of strokes avoided, and the cost per stroke avoided were estimated. During a 9-year horizon, the costs for the ICD and stroke treatment were €570 per patient-year for an ICD with atrial sensing capabilities and €491 per patient-year for a conventional ICD. Per 1,000 patients, 4.6 strokes per year are assumed to be avoided by the new device. Higher CHA2DS2-VASc scores are associated with higher numbers of avoided strokes and larger potential for cost savings. Apart from clinical advantages, the use of ICD with atrial sensing capabilities may reduce the incidence of stroke and, in high-risk patients, may also contribute to reduce overall health care costs.

## 1. Introduction

Implantable cardioverter-defibrillators (ICD) are indicated for the primary and secondary prevention of sudden cardiac death. Single-chamber ICD account for a significant proportion of ICD implanted today. In Germany, 38% of all implanted ICD during the year 2012 were single-chamber ICD [1]. Due to the missing atrial lead, conventional single-chamber ICD cannot provide atrial intracardiac electrograms (IEGM) as well as direct information about the atrial rate. This missing functionality is a significant limitation for the reliable detection of atrial tachyarrhythmias (ATs) including atrial fibrillation (AF). Nevertheless, AT/AF are frequent arrhythmias in patients with cardiac implantable electronic devices such as ICD or pacemakers. More over ICD patients have frequently clinical risk factors for stroke such as congestive heart failure, hypertension, advanced age, or previous myocardial infarction. A major indication (Class I) for an ICD implantation according to current ESC Guidelines is, e.g., the recommendation to reduce sudden cardiac death in patients with symptomatic heart failure (NYHA class II-III) and left ventricular ejection fraction ≤ 35% [2]. This recommendation includes patient subgroups with or without ischemic aetiology.

Two large prospective clinical trials (ASSERT and TRENDS) showed that device-detected ATs were associated with an increased risk of ischemic stroke [3, 4]. A pooled data analysis of five prospective studies with more than 10,000 patients confirmed the association between the device-detected AF burden and the increased risk of ischemic stroke [5]. In addition, a previous study has demonstrated that more than 90% of the device-detected AT/AF episodes are asymptomatic [6]. The ASSERT study showed that episodes of subclinical atrial tachyarrhythmia's occurred almost eight times more often than episodes of clinical AF [3].

Oral anticoagulation (OAC) in patients with nonvalvular AF is an effective therapy to reduce the risk of stroke and mortality [7]. In the 2016 ESC Guidelines for the management of atrial fibrillation [8], OAC therapy is a Class I and IIa recommendation for the management of AF in male patients with a CHA2DS2-VASc score ≥2 (≥3 for female patients) and 1 (2 for female patients), respectively. DeCicco et al. have provided a rationale for the use of long-term OAC therapy in patients with AF detected by implantable devices [9]. The current ESC Guidelines for the management of atrial fibrillation recommend interrogating pacemakers and implanted devices on a regular basis for atrial high rate episodes (AHRE) [8]. Patients with AHRE should undergo further assessment of risk factors and for overt AF. OAC therapy is recommended, e.g., if the following conditions are fulfilled: device patients present with AHRE (>5-6 min and >180 bpm), patients are eligible for OAC using CHA2DS2-VASc score, and AF is confirmed by review of device electrograms [8].

The Lumax VR-T DX system (Biotronik, Berlin, Germany) is a single-lead ICD with a floating atrial sensing dipole. The DX system includes the specifically designed Linox DX lead (Biotronik, Berlin, Germany) which combines the standard defibrillation and ventricular pacing function with atrial sensing. The Linox DX lead can be implanted like a standard single-chamber ICD. There are two versions of the lead (distance lead tip to atrial dipole: 150 mm and 170 mm) available. The accurate detection of the atrial rhythm is based on the sensing of atrial signals by the floating atrial dipole of Linox DX and the amplification of these signals by a modified atrial input stage of Lumax DX. Clinical data with the first generation of this ICD demonstrated the feasibility of atrial sensing during sinus rhythm as well as during arrhythmias such as AF [10, 11]. Data about the clinical efficacy, safety, and appropriate atrial sensing of the new generation Lumax VR-T DX ICD system were published by Safak et al. in 2013 [12]. The results demonstrated that the device reliably detected AF and provided corresponding atrial IEGMs.

The combination of ICD technology with an automatic home monitoring function allows for a significant rise in early detection of AF by a physician compared with conventional care [13]. Compared with other devices, the major difference in the DX system is that it provides additional atrial information which can be used for arrhythmia discrimination algorithms and AF detection. Based on the high atrial rate detection, the Lumax VR-T DX provides daily information about the 24-hour atrial tachyarrhythmia burden. The DX technology offers the opportunity to initiate OAC shortly after the occurrence of relevant AF episodes, thus potentially decreasing the AF-related stroke incidence.

Based on the above-mentioned considerations, the objective of the present model-based analysis was to measure the impact on costs due to reduced stroke frequency after implantation of the Lumax VR-T DX system compared to a conventional single-chamber ICD (Lumax VR-T, Biotronik, Berlin, Germany), which is very similar in basic ICD functionality to the Lumax VR-T DX system including home monitoring function. The model assumptions regarding OAC therapy to prevent strokes in patients with AF are based on current guidelines [8]. The primary outcome of our analysis was the difference in annual stroke-related costs per patient during a period of 9 years after de novo ICD implantation using the new single-chamber ICD system with atrial sensing capabilities compared to conventional ICD. Secondary outcomes were the determination of the expected mean annual number of strokes per group, the resulting mean annual number of strokes avoided, and the number needed to treat (NNT) for avoiding one stroke, resp., fatal stroke.

## 2. Methods

### 2.1. Model Design

**Figure 1:**
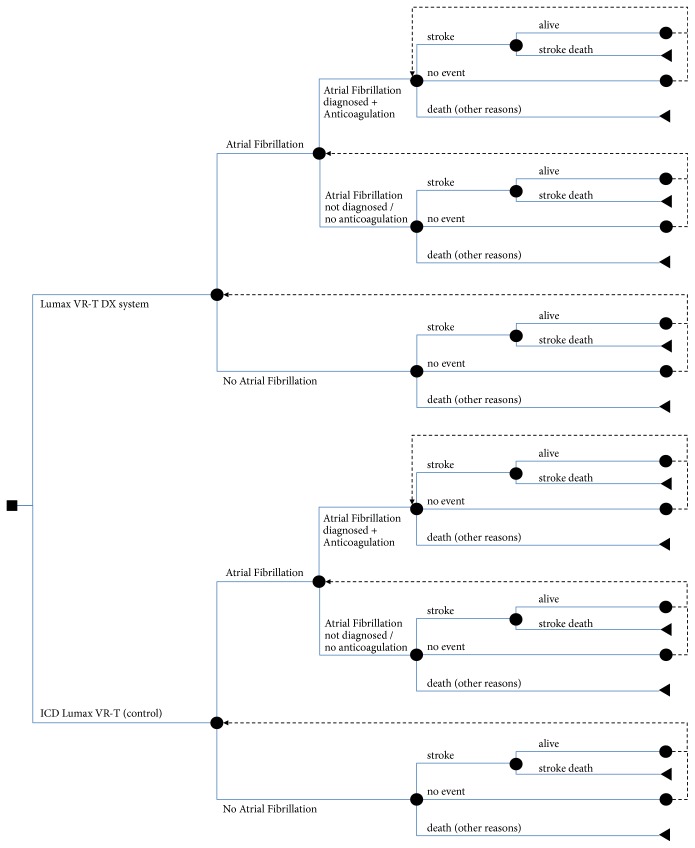
Model structure.

The study was designed as a health economic model calculation that considered different health states. Allowing for certain methodological restrictions, the path through these states was reflected by using a Markov cycle tree (Figure 1). A fictive cohort of 1,000 patients each was included into the model allowing the calculation of the number of patients in each of the predefined states in future years. Consequently, it was possible to calculate the expected number of strokes and stroke mortality and the associated costs.

The main assumption of the model was that patients with indication for a device could receive either a conventional single-chamber ICD Lumax VR-T (control) or the Lumax VR-T DX system. After the ICD implantation, both treatment groups had an equal probability of experiencing an AF episode since the Lumax VR-T DX system does not influence the AF incidence. Seidl et al. [14] demonstrated that the monitoring of a high atrial rate episode can be used for the reliable detection of atrial tachycardias (sensitivity of up to 98%). An AT/AF monitoring zone with IEGM recording based on high atrial rate detection can also be programmed in the Lumax VR-T DX, and it was assumed that the detection of ATs/AF can be realized with a high sensitivity (98%). False positive detection of atrial arrhythmias, e.g., due to atrial oversensing or far-field sensing is minimized by the specific input stage of the DX system with a dynamic adaptation of the atrial sensing threshold. Safak et al. [12] reported a high rate of 93.8% appropriate atrial sensing based on prespecified sensing tests. Moreover the physician can check the false positive detection by evaluating the atrial and ventricular IEGM which is provided for recorded AF episodes. A study presented by Sticherling et al. confirmed the diagnostic accuracy of the DX system in patients with permanent AF [15]. Particularly, 99% of the atrial IEGM allowed for a proper diagnosis of the atrial rhythm.

After AF detection, the model further assumed an immediate OAC treatment onset (for male patients CHA2DS2-VASc score ≥ 1, for female patients CHA2DS2-VASc score ≥ 2) either with Warfarin or with new oral anticoagulant (NOAC) Rivaroxaban, which is the most prescribed NOAC in Germany [16]. Patients with undetected AF would not receive any anticoagulation. According to the recent findings from the NORDIC ICD randomized clinical trial, we supposed a proportion of female patients of 19% [17]. For OAC we assumed that about 47.9% of detected AF patients receive Warfarin, 52.1% receive Rivaroxaban. This equals recent findings from the Global Anticoagulant Registry in the FIELD-Atrial Fibrillation on the distribution of Vitamin K Antagonists and NOACs in OAC of patients with newly diagnosed atrial fibrillation [18]. In the present model, OAC with Warfarin leads to a mean reduction of the stroke risk by 64% [7] and a reduction in mortality by one-third [19] compared to Placebo or no treatment. Assuming NOACs are slightly more effective in reducing strokes than Warfarin [20], we considered a stroke risk reduction of 66% for Rivaroxaban compared to untreated AF patients.

The annual probability for an untreated AF patient to experience a stroke event was considered for different patient risk groups depending on individual CHA2DS2-VASc scores [21]. For the base-case scenario, we calculated the annual stroke risk as a weighted combined risk according to the real world distribution of CHA2DS2-VASc scores observed in the German Heart Centre in Munich (N=171 patients, mean CHA2DS2-VASc score of 3.3; mean stroke risk of 3.96% per year; see Table 1). For patients with detected AF episodes receiving OAC, we assumed an annual risk for major bleeding of 3.4% under Warfarin and 3.6% for Rivaroxaban compared to 2.95% in patients without OAC [20, 22].

Further transition probabilities among the predefined health states were obtained either directly or derived from published literature sources (Table 2). The Markov model used in the present analysis was configured as Markov chain model assuming constant transition probabilities over time. One Markov cycle length was defined as one year. The total model duration was nine years, reflecting the mean duration of the ICD battery life.

For modeling, Microsoft Excel 2010 v14.0 was used. Future costs as well as effects (beginning from year two after implantation) were discounted by a mean annual rate of 3%, varied from 2% to 4% in additional sensitivity analyses (SA).

### 2.2. Cost Determination

The costs analysis was conducted from the perspective of the German statutory health insurance (SHI). To determine the total costs for each treatment cohort during the model duration, results on the number of patients in each health state per year were multiplied by the mean annual costs associated with each state derived from the published literature and supplementary assumptions (Table 3). Based on these results, the average annual cost per patient was predicted as follows:

(1) Ø  annual  costpatientICDLumax  VR-T  (control)  /Lumax  VR-T  DX  system=∑i=19cost  of  the  cohort  tinumber  of  patients  ti×19  years

Costs were restricted to stroke-related inpatient (acute and rehabilitation) and outpatient costs as well as costs due to acetylsalicylic acid (ASA) use. If patients with undetected AF suffered from stroke, we assumed they will receive ASA as standard medication. Costs for OAC were considered according to the kind of treatment. OAC with Warfarin were calculated as €65.70 per year, while Rivaroxaban is more costly with €1,241.00 per year. Additionally costs for major bleeding complications were involved as €1,995 per major bleeding event taken from Bufe at al. [34]. Remote monitoring costs were not considered as these costs were incurred in both groups. Implantation costs for ICD were also neglected since, for the implantation of the Lumax VR-T DX system, the same DRGs as for conventional ICD are reimbursed by statutory health insurances in Germany.

### 2.3. Sensitivity Analyses

To account for uncertainty in the model calculation, we conducted a deterministic as well as a probabilistic sensitivity analysis (SA). For the deterministic SA the model inputs were consecutively varied within realistic and predefined minimum-maximum ranges and the effect on the primary outcome (cost-difference between the groups) was observed. The cost-difference calculated for base-case scenario (over all CHA2DS2-VASc scores) was used as the reference. Inputs with a significant impact on the model results lead to a larger deviation from the base-case result and are potential sources for model uncertainty. Additionally we conducted a probabilistic SA based on a Monte-Carlo Simulation process [36] which involves running the model 1,000 times using randomly sampled values of all model inputs simultaneously. The random sampling was based on the predefined data ranges as used for deterministic SA and was conducted according to the nature of input data. For the cost data, a gamma-distribution was assumed while other data were assumed to be normally distributed. Afterward all 1,000 results were plotted into a cost-effectiveness plane which gives graphical information on the models robustness.

## 3. Results

### 3.1. Primary Outcome

**Figure 2:**
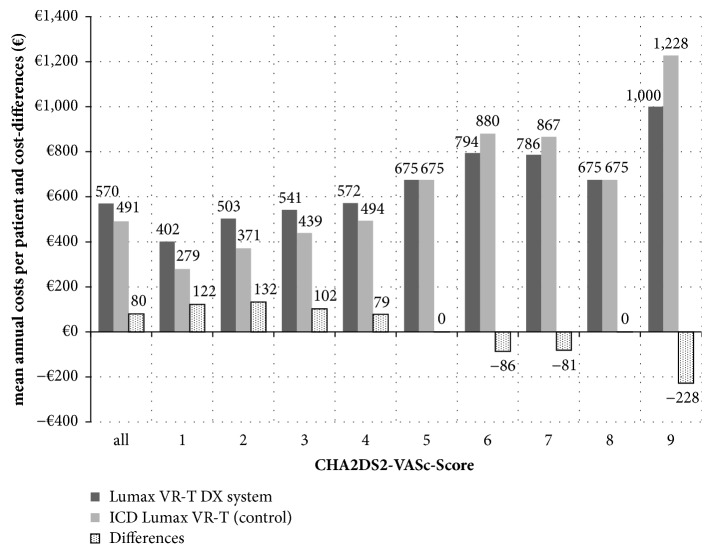
Mean annual costs per patient by group and cost-differences depending on CHA2DS2-VASc score.

Not considering the hospitalization costs related to the initial ICD implantation (which arise equally in both groups from SHI perspective), the mean patient-related costs during the first year were €337 for patients receiving the Lumax VR-T DX system compared to €295 for patients receiving the conventional ICD. The annual mean costs over a total duration of nine years (until device replacement) were €570 for Lumax VR-T DX and €491 for controls, resulting in slightly additional annual costs of about €80 per year (Figure 2).

Since the stroke risk strongly depends on the patient population, different results were observed with respect to the CHA2DS2-VASc scores, with growing benefit for patients with increasing CHA2DS2-VASc scores (and an increased risk of suffering from strokes). If a patient cohort with a CHA2DS2-VASc score of 2 receive a Lumax VR-T DX, the annual additional costs over a 9-year time horizon were expected to be at mean, merely €132, while the implantation of the device in a patient cohort with high risk for stroke (CHA2DS2-VASc score of 9) was expected to result in annual mean cost savings of €228. The additional costs for patients with the Lumax VR-T DX are primarily explainable by the higher proportion of patients receiving OAC after AF was detected.

### 3.2. Secondary Outcome

**Figure 3:**
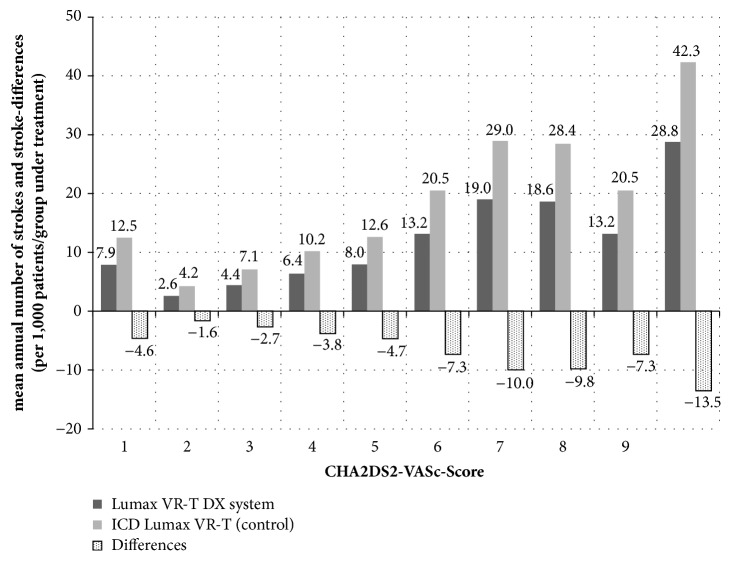
Mean annual number of strokes and stroke-differences depending on CHA2DS2-VASc scores (per 1,000 patients/group under treatment).

The earlier detection of patients suffering from AF and the related OAC onset was found to have a strong influence on the stroke incidence. In our base-case analysis, the mean annual number of strokes per 1,000 patients who underwent Lumax VR-T DX implantation was 7.9 versus 12.5 for controls (Figure 3). The resulting number of 4.6 stokes avoided per year was the main reason for the observed cost savings. As with the economic results, we observed a wide difference depending on the patient's clinical characteristics. The impact on stroke incidence was the expectable lowest in patients with a CHA2DS2-VASc score of 1 (1.6 avoided strokes per year) and increased with higher risk (CHA2DS2-VASc score of 9, 13.5 avoided strokes per year). The NNT for avoiding one stroke per year ranged from 617 (CHA2DS2-VASc score of 1) to 74 (CHA2DS2-VASc score of 9), the NNT for avoiding one stroke-related death ranged from 800 (CHA2DS2-VASc score of 1) to 89 (CHA2DS2-VASc score of 9).

### 3.3. Sensitivity Analyses

**Figure 4:**
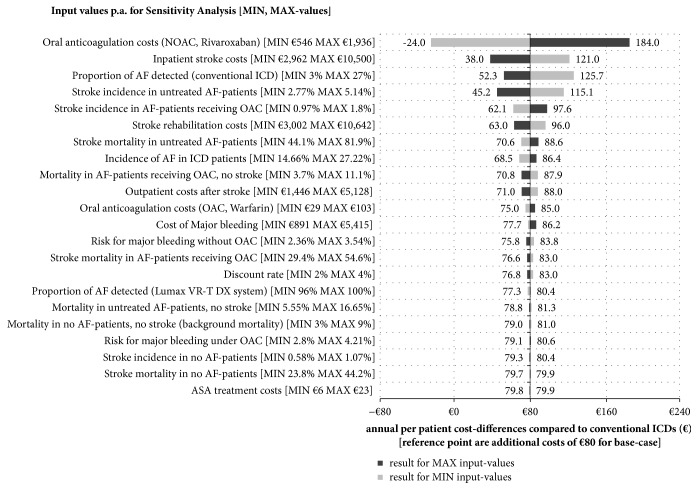
Results for deterministic sensitivity analysis (for base-case): mean annual per patient cost-difference under consecutively varying minimum and maximum input-values.

Our results were proved to be robust in additional SA for the base-case. The results of our deterministic SA indicate the following variables which had the largest impact on the model robustness: cost of NOAC treatment, inpatient stroke costs, and the proportion of detected AF as well as stroke incidence (Figure 4). Nevertheless, after varying these inputs with minimum and maximum values, the main conclusion of the study keeps untouched. For example, we assumed a mean AF-detection rate of 15% in conventional ICD in our base-case analysis, resulting in additional costs of €80 for Lumax VR-T DX. In the deterministic SA this detection rate was replaced by ±80% with a minimum value of 3% and a maximum value of 27%, resulting in different additional costs for Lumax VR-T DX of €126 and €52, respectively.

**Figure 5:**
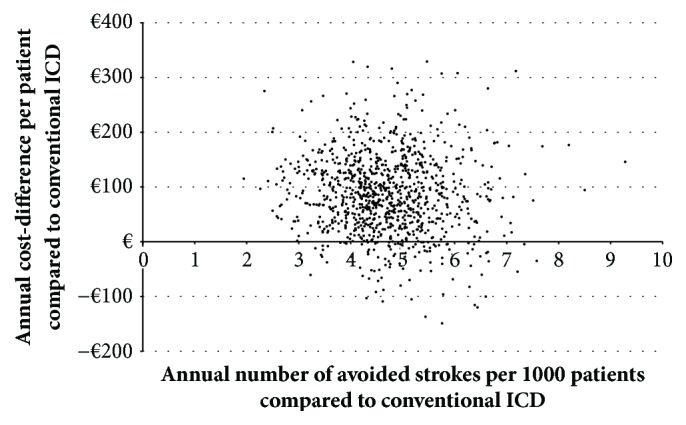
Results for probabilistic sensitivity analysis (for base-case): mean annual per patient cost-difference and number of avoided strokes per 1,000 patients under treatment under randomly and simultaneously varying input-values.

In our probabilistic SA all of the 1,000 random model results were plotted into a cost-effectiveness plane (Figure 5). As most of the single dots (each dot for each model run) are located in the upper right quadrant, it seems that our results are reasonably robust, especially with regard to our findings on stroke avoiding, where 100% of our model results are laying right to the y-axis. The probability to observe a cost saving effect due to Lumax VR-T DX implantation was 11.3% for base-case, ranging from 0% for patients with a CHA2DS2-VASc score of 1 to 93.2% for patients with a CHA2DS2-VASc score of 9.

## 4. Discussion

To our knowledge, our study is the first analysis to present the economics as well as the effectiveness of a single-chamber ICD with additional atrial sensing capabilities (Lumax VR-T DX). It should be considered as a pilot study providing early information and help in the decision-making process determining which type of patients would benefit most from using this system or for planning prospective studies. The simulation in the present analysis indicates that the use of the Lumax VR-T DX system, accompanied by an earlier OAC onset, would lead to a reduction of the number of strokes. Our analysis is noteworthy in that we have included and evaluated some important characteristics (different stroke risk groups through the use of CHA2DS2-VASc scores) that offer more detailed information. Additionally, we conducted sensitivity analyses to obtain numerical results and to determine the parameters of uncertainty transparently. The main results of our base-case analysis are likely to reflect the real world setting since we took into account the distribution of CHA2DS2-VASc scores among patients of a real existing high volume ICD implantation center.

Beside these strengths, our analysis has a number of limitations that should be discussed. First, our model assumes frequencies of relevant risk factors such as diabetes or arterial hypertension to be constant over time. In fact, there will be some patients who newly develop these risk factors and therefore classify in a higher CHA2DS2-VASc scores over time. This is not reflected in our model and therefore the costs may be underestimated. Another reason, reflecting a more conservative character of our model calculation can be found in the predefined health insurance perspective. As a consequence, the model is only focused on direct cost consequences; the influence on indirect cost was not analyzed. Furthermore, the use of CHA2DS2-VASc scores, to get a measure for stroke risk per year, should be discussed. The CHA2DS2-VASc score combines the number of single risk factors and their influence on annual stroke incidence, while higher score is associated with increased stroke risk. Interestingly our model shows comparatively similar results for the cohort of patients with CHA2DS2-VASc score of 5 and a score of 8 (see Figures 2 and 3). This observation is explainable by the underlying adjusted annual stroke risk, which is given to be 6.7% for both score values [23]. The stroke risk of patients with ICD or pacemakers depends on different variables such as occurrence of device-detected AF, duration, and frequency of AF and individual risk factors [9]. Analogous to the 2016 ESC Guidelines for the management of atrial fibrillation a scheme considering 2 criteria (occurrence of device-detected AF episodes and CHA2DS2-VASc score) was proposed for the decision to initiate OAC in device patients [8]. We did not distinguish between short and clinical relevant AF episodes since it is still unclear whether AHRE imply the same therapeutic requirements as overt AF [7]. Furthermore, we did not include extra costs for OAC use with Warfarin such as regular international normalized ratio monitoring or additional outpatient visits. The assumptions in our model regarding the effect of stroke prevention by OAC and the bleeding risk are based on studies not specifically considering patients with device-detected AF but studies with patients suffering from nonvalvular clinical AF [7, 20, 22]. So a further limitation of our model is a possible overestimation of the effect to reduce the stroke risk. Although for ICD patients the ideal antithrombotic and anticoagulation therapy has not yet been settled [37], we included an OAC treatment mix with Warfarin as well as with Rivaroxaban to reflect the routine care. NOACs have recently entered the market, and are prescribed with increasing frequency as they are recommended as a possible first line therapy in patients with nonvalvular atrial fibrillation [8]. In our study we assumed a similar proportion of Warfarin/NOAC use as it was investigated in a nonvalvular AF population [18]. This assumption can be discussed, since many ICD recipients have concomitant coronary artery disease and may present with acute coronary syndrome or the need for percutaneous coronary interventions. Furthermore the consideration of NOACs is a major reason for the additional costs of Lumax VR-T DX patients detected in our model, because NOACs are markedly more expensive compared to Warfarin. An alternative base-case calculation only considering Warfarin as an OAC treatment option would lead to an overall cost saving for patients with Lumax VR-T DX of €95 compared to conventional ICD. Since we referred in our model to the 2016 ESC Guidelines for the management of atrial fibrillation, we did not take into account cost-effectiveness measurement in a model which is based on a possible future concept of OAC therapy in patients without documented AF but with elevated stroke risk. So a study by Tischer et al. found that, in patients with high CHA2DS2VASc-scores, thromboembolic complications occurred irrespective of the presence of AF and concluded that anticoagulant therapy may be initiated irrespective of documented AF [38]. But superiority of this new concept for prevention of stroke has not been proven so far.

Compared with the single-chamber ICD, one further potential benefit of the Lumax VR-T DX system is the reduced number of inappropriate therapies, such as inappropriate shocks, through the use of the enhanced Biotronik arrhythmia SMART detection algorithm. In addition to the sudden ventricular rate increase and stability criterion, the SMART algorithm analysis also provides atrial rate information for enhanced arrhythmia discrimination which may reduce inappropriate therapies and increase device as well as battery longevity [39]. These corresponding cost factors in favor of the Lumax VR-T DX were also not considered in our model.

The actual German DRG System does not reflect the described clinical benefit and, therefore, the treatment with the DX ICD is currently reimbursed as a conventional single-chamber system which is accompanied by lower material costs from hospitals perspective. To enable a wider clinical application of the DX technology and to gain the described benefit for the patients, this economic disadvantage for hospitals could be discussed.

## 5. Conclusion

The implantation of the Lumax VR-T DX system appears to be associated with lower stroke incidence but additional costs for the statutory health insurance over the battery lifetime. These additional costs are explainable due to the higher number of detected AF patients and the subsequent OAC onset. However, the cost impact is strongly influenced by the underlying stroke risk of the population under treatment, with the growing likelihood for cost savings with increased CHA2DS2-VASc scores.

## Figures and Tables

**Table 1:** Distribution of CHA2DS2-VASc score in single chamber ICD recipients without indication for anticoagulation (171 patients; mean age 59.1 (SD±15.5) years) from 2011/01/01 to 2013/10/31, Heart Centre in Munich) used for calculation of the base-case scenario stroke rate; adjusted stroke rate per year [23].

**CHA2DS2-VASc Score**	**n (%)**	**Adjusted stroke rate per year**
0	13 (7.6)	0%
1	16 (9.4)	1.3%
2	28 (16.4)	2.2%
3	31 (18.1)	3.2%
4	40 (23.4)	4.0%
5	27 (15.8)	6.7%
6	13 (7.6)	9.8%
7	2 (1.2)	9.6%
8	1 (0.6)	6.7%
9	0 (0.0)	15.2%
All (base-case)	171 (100.0)	3.96% (combined risk set as base-case)

Frequency of CHA2DS2-VASc risk factors in total sample: congestive heart failure = 87.13%; hypertension = 70.18%; age > 75 = 25.73%; diabetes mellitus = 22.22%; stroke/TIA = 31.58%; vascular disease = 9.36%; age: 65-74 = 58.48%; sex category: women = 15.79%.

**Table 2:** Annual transition probabilities.

**Model item **	**Mean annual probability **	**Sources/underlying assumptions**
Incidence of AF in ICD patients	20.94%	Own calculation based on publications by Safak et al. 2013 [12], Bunch et al. 2009 [24], Mittal et al. 2008 [25] and Healey et al. 2012 [3]

Proportion of AF detected (Lumax VR-T DX system)	98.00%	Derived from Seidl et al. 1998 [14] Recording of atrial tachyarrhythmias based on high atrial rate detection.

Proportion of AF detected (conventional ICD)	15.00%	AF episodes recording based on single-chamber ICD detection criteria. Confirmation of AF by 24-hour Holter monitoring. Own calculation based on publications by Friedmann et al. 2006 [26], Moss et al. 2012 [27] and Charitos et al. 2012 [28]

Stroke incidence in untreated AF-patients	Based on CHA2DS2VASc, for base case: 3.96%	Directly derived according to the CHA2DS2-VASc-Score. For the base-case scenario, a weighted incidence was calculated according to the distribution of CHA2DS2-VASc-Scores found in German Heart Center Munich

Stroke incidence in no AF-patients	for base case: 0.82%	According to Wolf et al. 1991 [29], the risk of stroke is 4.8 fold increased if a patient is suffering from AF. In reversing circuit, the stroke incidence in patients without AF is the stroke incidence in untreated AF patients / 4.8

Stroke incidence in AF-patients receiving OAC	for base case: 1.38%	According to Hart et al. 2007 [7], anticoagulation using Warfarin is associated with a mean stroke risk reduction of 64% (49% to 74%). Stroke incidence in AF patients receiving Warfarin was calculated as follows: Stroke incidence in untreated AF patients *∗* (1-0.64). Since NOACs are slightly more effective in reducing strokes than Warfarin (Patel at al. 2011 [20]), we considered a stroke risk reduction of 66% for NOACs compared to untreated AF patients. The stroke incidence used in the model for OAC was calculated as a weighted mean according to the distribution of NOACs and Warfarin (52.1% vs. 47.9%).

Stroke mortality in no AF-patients	34.00%	Directly derived from Lin et al. 1996 [30] where 41 of 120 non AF patients died within one year

Stroke mortality in untreated AF-patients	63.00%	Directly derived from Lin et al. 1996 [30] where 19 of 30 AF patients died within one year

Stroke mortality in AF-patients receiving OAC	42.00%	According to Lip et. al. 1996 [19] where anticoagulation with warfarin reduced mortality by a third. Stroke mortality in AF patients receiving anticoagulation was calculated as follows: Stroke mortality in untreated AF patients *∗* (1-1/3)

Mortality in no AF-patients, no stroke (background mortality)	6.00%	Directly derived from van Welsenes et al. 2011 [31]. The cumulative incidence for all-cause mortality was 6% at year 1.

Mortality in untreated AF-patients, no stroke	11.10%	Own calculation based on Stewart et al. 2002 [32] who reported a mortality increase of RR=2.2 in women and 1.5 in men. We assumed a mean mortality RR for both sexes of 1.85. This was multiplied with background mortality of 6%

Mortality in AF-patients receiving OAC, no stroke	7.40%	According to Lip et al. 1996 [19] where anticoagulation with Warfarin reduced mortality by one third. Mortality in AF patients receiving anticoagulation was calculated as follows: Mortality in untreated AF patients *∗* (1-1/3)

Risk for major bleeding under OAC	3.50%	Directly derived from ROCKET AF study (Patel et al. 2011 [20]) where the event rate of major bleeding was 3.4 per 100 patient-years for patients with nonvalvular AF and treatment with Warfarin. The corresponding bleeding rate under Rivaroxaban was reported as 3.6. The risk for major bleeding used in the model was calculated as a weighted mean according to the distribution of NOACs and Warfarin (52.1% vs. 47.9%).

Risk for major bleeding without OAC	2.95%	Own calculation based on Go et al. 2003 [22] where the crude rate of major hemorrhage was 1.52 events (1.28 events) per 100 patient-years in a real world patient population with nonvalvular AF receiving anticoagulation (resp. not receiving anticoagulation). Only events that did lead to hospitalization were analyzed in this study. This may have been associated with a risk of underreporting of events. Therefore, we multiplied the estimated reduction of major bleeding events (1.28/1.52) without OAC with the rate of major bleeding events under OAC used in the present model.

AF: atrial fibrillation; ICD: implantable cardioverter-defibrillator; NOACs: new oral anticoagulants; OAC: oral anticoagulation; RR: relative risk.

**Table 3:** Annual costs according to health state.

**Cost factor**	**Mean costs per year **	**Underlying calculations** **∗**
Costs per AF-patient receiving OAC, stroke, alive	€17,518	100% *∗* inpatient stroke costs + 100% *∗* stroke rehabilitation costs + 100% *∗* outpatient costs after stroke hospitalization + 100% *∗* oral anticoagulation costs

Costs per AF-patient receiving OAC, fatal stroke	€11,852	100% *∗* inpatient stroke costs + 46% (proportion of patients with at least 4 weeks survival) *∗* stroke rehabilitation costs + 50% *∗* outpatient costs after stroke hospitalization + 50% *∗* oral anticoagulation costs

Costs per AF-patient receiving OAC, no stroke, alive	€678	100% *∗* oral anticoagulation costs

Costs per AF-patient receiving OAC, no stroke, death	€339	50% *∗* oral anticoagulation costs

Costs per AF-patient (not detected, no OAC), stroke, alive	€19,143	100% *∗* inpatient stroke costs *∗* 1,34 (cost increase due to untreated AF [33]) + 100% *∗* stroke rehabilitation costs + 100% *∗* outpatient costs after stroke + 100% *∗* costs ASA treatment

Costs per AF-patient (not detected, no OAC), fatal stroke	€13,808	100% *∗* inpatient stroke costs *∗* 1,34 (cost increase due to untreated AF [33]) + 46% (proportion of patients with at least 4 weeks survival) *∗* stroke rehabilitation costs + 50% *∗* outpatient costs after stroke + 50% *∗* costs ASA treatment

Costs per AF-patient (not detected, no OAC), no stroke, alive	-	No costs considered

Costs per AF-patient (not detected, no OAC), no stroke, death	-	No costs considered

Cost per no AF-patient, stroke, alive	€16,855	100% *∗* inpatient stroke costs + 100% *∗* stroke rehabilitation costs + 100% *∗* outpatient costs after stroke hospitalization + 100% *∗* costs ASA treatment

Cost per no AF-patient, fatal stroke	€11,520	100% *∗* inpatient stroke costs + 46% (proportion of patients with at least 4 weeks survival) *∗* stroke rehabilitation costs + 50% *∗* outpatient costs after stroke hospitalization + 50% *∗* costs ASA treatment

Cost per no AF-patient, no stroke, alive	-	No costs considered

Cost per no AF-patient, no stroke, death	-	No costs considered

Cost of Major bleeding	€1,995	Mean attributable costs due to major bleedings according to Bufe et al. 2009 [34] (ranging from €891 to €5,415)

For deceased patients, we assumed the costs for medications and outpatient treatment for a half year (50%).

*∗*Annual unit costs used for calculations above: inpatient stroke costs €6,731 [35], stroke rehabilitation costs €6,822 [35], outpatient costs after stroke hospitalization €3,287 [35], oral anticoagulation costs €65.70 for Warfarin and €1,241.00 for Rivaroxaban [16] (weighted mean: €678.18), and costs of ASA treatment €14.60 [16].

ASA: acetylsalicylic acid; AF: atrial fibrillation; OAC: oral anticoagulation.

## Data Availability

The data used to support the findings of this study are included within the article.
